# Cross-national differences in stroke management in the Baltic states: analysis within the Stroke Action Plan for Europe framework

**DOI:** 10.1093/esj/aakag050

**Published:** 2026-05-27

**Authors:** Rytis Masiliūnas, Edgaras Zaboras, Vitalija Šumskienė, Kristaps Jurjāns, Janika Kõrv, Riina Vibo, Evija Miglāne, Guntis Karelis, Dalius Jatužis, Aurelija Daškevičiūtė, Hanne Christensen, Aleš Tomek, Francesca Romana Pezzella, Aleksandras Vilionskis

**Affiliations:** Clinic of Neurology and Neurosurgery, Institute of Clinical Medicine, Faculty of Medicine, Vilnius University, Vilnius, Lithuania; Center for Digital Medicine, Translational Health Research Institute, Faculty of Medicine, Vilnius University, Vilnius, Lithuania; Center for Digital Medicine, Translational Health Research Institute, Faculty of Medicine, Vilnius University, Vilnius, Lithuania; Department of Neurology, Republican Vilnius University Hospital, Vilnius, Lithuania; Department of Neurology and Neurosurgery, Riga Stradiņš University, Riga, Latvia; Department of Medicine, The Red Cross Medical College of Riga Stradiņš University, Riga, Latvia; Neurology Department, Pauls Stradiņš Clinical University Hospital, Riga, Latvia; Department of Neurology and Neurosurgery, University of Tartu, Tartu, Estonia; Department of Neurology and Neurosurgery, University of Tartu, Tartu, Estonia; Department of Neurology and Neurosurgery, Riga Stradiņš University, Riga, Latvia; Neurology Department, Pauls Stradiņš Clinical University Hospital, Riga, Latvia; Department of Neurology and Neurosurgery, Riga Stradiņš University, Riga, Latvia; Research Professor (Tenured Professor) Group in Neuroimmunology, Department of Biology and Microbiology, Rīga Stradiņš University, Riga, Latvia; Clinic of Neurology and Neurosurgery, Institute of Clinical Medicine, Faculty of Medicine, Vilnius University, Vilnius, Lithuania; Clinic of Neurology and Neurosurgery, Institute of Clinical Medicine, Faculty of Medicine, Vilnius University, Vilnius, Lithuania; Department of Neurology, Copenhagen University Hospital Bispebjerg, Copenhagen, Denmark; Department of Neurology, Second Faculty of Medicine, Charles University and Motol University Hospital Motol, Prague, Czech Republic; Stroke Unit, Department of Neuroscience, San Camillo Forlanini Hospital, Rome, Italy; Clinic of Neurology and Neurosurgery, Institute of Clinical Medicine, Faculty of Medicine, Vilnius University, Vilnius, Lithuania

**Keywords:** Baltic states, recanalisation therapy, stroke care quality, stroke care systems, stroke services

## Abstract

**Introduction:**

Although epidemiological studies often group the Baltic states together, they differ significantly in national stroke care legislation and infrastructure. Our study aimed to explore and compare the current state of stroke care in Lithuania, Latvia and Estonia.

**Patients and methods:**

We analysed the Stroke Action Plan for Europe (SAP-E) Stroke Service Tracker data from 2022, including data from the respective National Health Insurance Funds and direct centre-level queries. Geographic Information System-based modelling assessed population access to stroke-ready hospitals within 1 h. Key metrics, including hospitalised stroke incidence, stroke unit admission, recanalisation therapy and in-hospital as well as 30-day mortality, were compared using Z-tests for proportions.

**Results:**

The hospitalised stroke incidence per 100,000 inhabitants was similar in Lithuania (353) and Latvia (354), but lower in Estonia (246), despite similar population structures. Lithuania had the highest proportion of its population (94.0%) with access to a stroke-ready hospital within 1 h, followed by Latvia (87.1%) and Estonia (84.7%, *P* < .001). Estonia had the highest proportion of stroke unit admission rates and the lowest mortality rates—9.6% (in-hospital) and 15.0% (30-day) for ischaemic stroke. Endovascular treatment was most frequent in Lithuania (8.6% of all strokes, *P* < .001), while Estonia had the highest rate of intravenous thrombolysis (29.0%, *P* < .001).

**Conclusions:**

Despite broadly comparable populations and formal SAP-E alignment, the Baltic states exhibit marked differences in stroke access, treatment and outcomes. High stroke unit admissions and high recanalisation rates in Estonia may be associated with lower ischaemic stroke mortality, underscoring the importance of system design beyond geographic coverage alone.

## Graphical Abstract

**Graphical Abstract:**
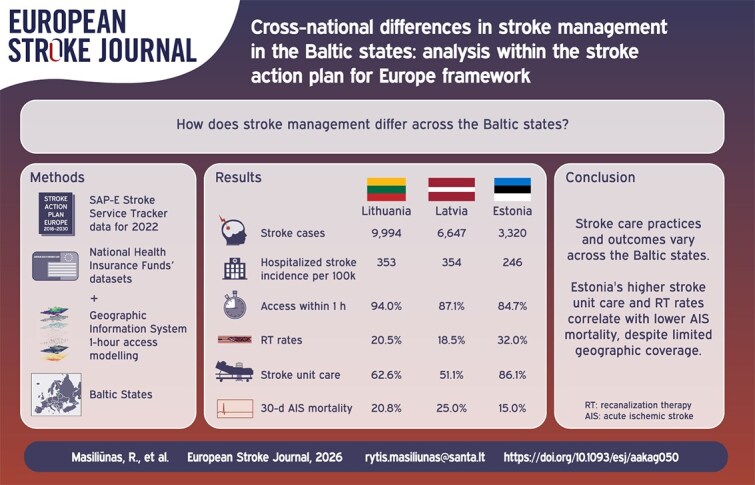

## Introduction

Stroke remains one of the leading causes of death and long-term disability in Europe, posing a significant public health challenge.^1–3^ Fortunately, stroke is a condition for which substantial prevention, treatment and rehabilitation strategies exist. With timely interventions and well-organised healthcare systems, it is possible to significantly reduce stroke incidence and improve outcomes for survivors.^4–6^ Yet, disparities in access to care and variability in service quality persist across regions, highlighting the need for unified and sustained efforts to improve stroke care throughout the continent.^7^

To address this need, 2 landmark pan-European consensus meetings—the Helsingborg Declarations of 1995 and 2006—were convened to assess the state of stroke services and establish strategic targets for improvement.^8^^,^^9^ Building on this foundation, the European Stroke Organisation (ESO), in partnership with the Stroke Alliance for Europe (SAFE), launched the Stroke Action Plan for Europe (SAP-E) for the period 2018–2030. The plan emphasises standardised data collection and cross-country comparisons to reduce disparities and guide national stroke care strategies.^6^ Stroke Action Plan for Europe also expands its focus beyond acute care to include primary prevention, life after stroke and research priorities, aiming for a comprehensive and equitable stroke care strategy across Europe.

To support these objectives, the SAP-E Stroke Service Tracker (SST) enables yearly reporting and comparison of key stroke care indicators across countries, utilising data provided by national coordinators.^10^ A mid-term review, based on 2022 data, has recently been published, reiterating notable inequities in access to care across Europe.^11^

The Baltic states—Lithuania, Latvia and Estonia—joined the SAP-E initiative to align their national stroke strategies with European benchmarks and to contribute to improving outcomes for stroke patients. While the political commitment to SAP-E is evident in all 3 countries, each faces specific challenges, including limited resources, regional inequalities in stroke service availability and gaps in long-term support for stroke survivors.^12–14^ Despite these hurdles, the Baltic states have demonstrated progress in areas such as access to intravenous thrombolysis (IVT) and EVT for patients with ischaemic stroke.^3^^,^^7^ However, few direct comparisons of stroke care across the Baltic states have been conducted to date, and full implementation of the SAP-E goals remains a work in progress.

Therefore, using the SAP-E SST data, our study compares the structure and performance of stroke-care systems in the 3 Baltic states, analysing current practices, service coverage and policy development to provide insights into their respective journeys towards meeting the 2030 SAP-E targets and pinpointing the gaps that require attention.

## Patients and methods

### Study design and data source

We analysed the data provided to SAP-E by the expert national coordinators (D.J., A.V., E.M., G.K., J.K. and R.V.) of each participating country, which were collected from several different sources. The data were intended to cover all recorded stroke cases in the Baltic countries for the full calendar year 2022. Respective National Health Insurance Funds were the data sources for stroke unit care admission, total number of ischaemic, haemorrhagic and unspecified stroke, subarachnoid haemorrhage admissions, their age (both median and mean), gender, number of different recanalisation therapies employed (IVT, EVT and bridging therapy) for ischaemic stroke patients, as well as stroke mortality at discharge and at 30 days.

In all 3 Baltic states, National Health Insurance Fund data are used to reimburse healthcare institutions for their services and provide a source for official health-related statistics, hospital performance evaluation and healthcare service planning. Reimbursement for active inpatient healthcare services is based on the Diagnosis-Related Groups method, and follows the International Statistical Classification of Diseases and Related Health Problems, 10th Revision (ICD-10). In Lithuania, ICD-10 Australian Modification (ICD-10-AM) is used, and the relevant medical interventions and treatments performed are coded using Australian Classification of Health Interventions (ACHI) codes.^15^ Data are collected through electronically submitted forms after patient discharge and cover all healthcare institutions, including non-stroke-ready and private hospitals, minimising the risk of underreporting patients treated outside stroke units or with severe strokes. Data collected are admission-based and may include recurrent strokes, and thus do not reflect true stroke incidence, but rather hospital admission rates (hospitalised stroke incidence). Nevertheless, across all 3 Baltic states, patients are not double-counted when transferred between hospitals, as the numbers are case-based, and eliminate duplicate registrations for a single episode.

All 3 National Health Insurance Funds have standardised data collection protocols, training and methodological materials for data abstractors (mostly physicians, but also specialised clinical coders), clinical coding quality reports and other multilevel data quality control measures.^15^ Validation typically includes planned, unplanned and periodic manual and automated checks for missing, inconsistent or medically implausible diagnosis and procedure combinations, as well as a manual review and correction when needed. The systems function primarily as claims-based administrative registries, not clinical registries. The methodology for obtaining the respective datasets and ensuring their completeness is available online.^16–18^

In addition, for data not available from these resources, individual contacts with sites were used to maximise data accuracy for the number of EVTs in Lithuania and Latvia, and IVT for Lithuanian ischaemic stroke patients. Finally, the SAP-E national coordinators estimated the number of beds in stroke units with IVT and EVT capability. The full interactive dataset is available online.^19^

### Setting

All 3 Baltic countries maintain compulsory National Health Insurance Funds that provide free access to state-sponsored medical services ranging from primary care and emergency medical services (EMS) to inpatient treatment, primary and secondary prevention programs, prescription medications and rehabilitation, among others. In each Baltic country, a nationwide network of stroke centres has been strategically positioned to ensure that patients can arrive within the critical treatment window for acute stroke interventions.^20^

**Figure 1:**
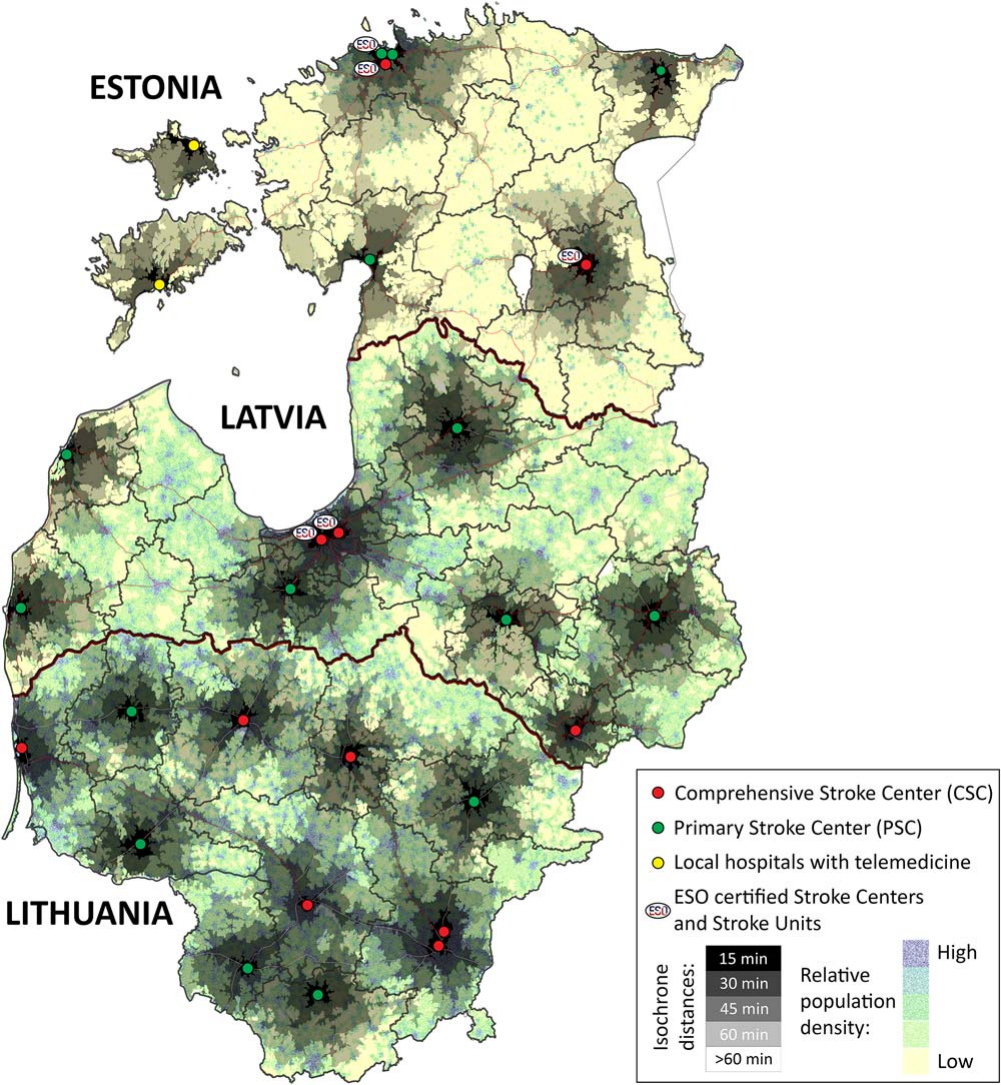
The distribution of Lithuanian, Latvian and Estonian primary and comprehensive stroke centres, along with isochrone distances to reach them, is superimposed on a relative population density map of the 3 Baltic states.

While Lithuania (65,300 km^2^), Latvia (64,600 km^2^) and Estonia (45,300 km^2^) share similar territorial extents, their population figures and densities differ markedly. Lithuania features 6 comprehensive stroke centres (CSCs) and 5 primary stroke centres (PSCs); Latvia operates 3 CSCs (2 operating around the clock) alongside 6 PSCs and Estonia maintains 2 CSCs and 4 PSCs, along with 2 additional local hospitals equipped with telemedicine. Local guidelines dictate that any person with suspected stroke must be taken to the closest stroke-ready hospital to guarantee rapid care. Physicians in all 3 Baltic countries can consult an on-call neurologist to determine whether a patient should be transferred to a CSC for EVT. Figure 1 displays the stroke-ready hospitals with isochrone contours showing Sunday evening car travel times, mapped using publicly available Google Maps data overlaid on each country’s relative population density.

### Ethics

The study was conducted in accordance with the guidelines of the Declaration of Helsinki. No ethics approval was required as the data are aggregated, anonymised and freely available online.

### Statistical analysis

We assessed the population accessibility to PSC and CSC within Lithuania, Latvia and Estonia. Isochrone maps were generated using the openrouteservice API, modelling travel time for emergency vehicles driving at maximum speeds in regular traffic flow to either a PSC or a CSC. High-resolution population distribution data for the 3 countries were sourced from WorldPop (2020). Spatial analysis was conducted using QGIS (version 3.40.0), where population data were overlaid with isochrone polygons to estimate the number of individuals reachable within a 1-h window. The Geographic Information System (GIS) accessibility modelling followed a deterministic approach, ensuring reproducible and internally consistent results due to the use of fixed isochrones and high-quality gridded population data. However, this approach does not eliminate uncertainty arising from modelling assumptions, such as travel speeds, routing choices and real-world emergency response conditions. As such, the resulting estimates represent deterministic point values derived from full population coverage rather than sampled data and do not incorporate stochastic variability. The statistical analysis was conducted using standard GIS-based analytical methods. Statistical comparisons between countries were performed using Z-tests for proportions, with accessibility defined as a binary outcome (reachable vs not reachable). The large population sizes satisfied the assumptions of independence and normal approximation required.

Hospitalised stroke incidence was calculated as the annual number of hospital-treated ischaemic stroke cases per 100,000 inhabitants. This measure reflects hospital-based stroke incidence rather than true incidence, as it may include recurrent events, omit pre-hospital deaths and capture only hospitalised patients. We divided the total number of recorded cases by the official population size for each country in the corresponding year. Categorical variables are reported as frequencies with percentages, while continuous variables are summarised as the mean with SD. The Shapiro–Wilk test was applied to assess normality. For comparisons across the 3 Baltic states, normally distributed continuous variables were analysed using one-way ANOVA. Associations between categorical variables were examined using the χ^2^ test or the Fisher–Freeman–Halton exact test when appropriate. All statistical tests were 2-sided, with a significance threshold set at *P* < .05. No imputation was performed for missing values. Analyses were performed on available cases only.

## Results

In 2022, Lithuania was the most populous of the Baltic states, with 2.8 million residents, followed by Latvia with 1.9 million and Estonia with 1.3 million. Access to stroke treatment varied among the countries, with Lithuania having the largest stroke care network. The proportion of the population with access to a stroke-ready hospital within 1 h ranged from 84.7% in Estonia to 94.0% in Lithuania, with a statistically significant difference in reachability between countries. Despite its smaller population, Estonia had a significantly higher proportion of stroke unit care admissions, amounting to 86.1% of cases, followed by Lithuania with 62.6% and Latvia with 51.1%. For comparison of hyperacute stroke care in the Baltic states, see Table 1.

**Table 1:** Comparison of stroke prevalence and hyperacute stroke care between the 3 Baltic states in 2022, based on National Health Insurance Fund data.

*n* (%)	Lithuania	Latvia	Estonia	*P* value
**Total population^a^**	2,831,639	1,879,383	1,348,840	
**Comprehensive stroke centres**	6	3	2	
**Primary stroke centres**	5	6	4 (+2)	
**Proportion of population with access to a stroke-ready hospital within 1 h, %**	94.0	87.1	84.7	<.001
**Number of beds in stroke units with IVT^b^**	150	58	90	
**Number of beds in stroke units with EVT^b^**	75	26	31	
**Stroke unit care admission**	6260 (62.6)	3398 (51.1)	2860 (86.1)	<.001
**All strokes**	9994	6647	3320	<.001
** Ischaemic stroke (IS)**	8643 (86.5)	5790 (87.1)	2892 (87.1)	.427
** Haemorrhagic stroke**	1266 (12.7)	857 (12.9)	422 (12.7)	.910
** Intracerebral haemorrhage (ICH)**	966 (9.7)	635 (9.6)	326 (9.8)	.910
** Subarachnoid haemorrhage**	300 (3.0)	222 (3.3)	96 (2.9)	.350
** Undetermined**	85 (0.9)	0	6 (0.2)	<.001
**Hospitalised stroke incidence per 100,000 inhabitants†**	353	354	246	<.001
**Median age of all stroke patients, years**	75	75	76	
**Mean age of all stroke patients, years**	73.0	73.2	74.4	
**Female gender**	5277 (52.8)	3563 (53.6)	1788 (53.9)	.443
**Recanalisation therapy for IS^c^**	1776 (20.5)	1073 (18.5)	927 (32.0)	<.001
** Intravenous thrombolysis (IVT)**	1284 (14.8)	974 (16.8)	839 (29.0)	<.001
** EVT**	748 (8.6)	217 (3.7)	222 (7.7)	<.001
** IVT and EVT**	256 (3.0)	118 (2.0)	134 (4.6)	.021
**Mortality at discharge, %**				
** Ischaemic stroke**	12.2	15	9.6	<.001
** Intracerebral haemorrhage**	24.9	37	30.3	<.001
**Mortality at 30 days**				
** Ischaemic stroke**	20.8	25	15.0	<.001
** Intracerebral haemorrhage**	39.7	51	41.7	<.001

Abbreviations: IS, ischaemic stroke; IVT, intravenous thrombolysis.

^a^Stroke Action Plan for Europe data (or Geographic Information System).

^b^Estimate.

^c^Individual contact to site (except for Estonia).

Compared to population size, Latvia and Lithuania had nearly identical hospitalised stroke incidence rates in 2022, with 354 and 353 cases per 100,000 inhabitants, respectively, while Estonia had a significantly lower rate at 246 per 100,000. Demographic characteristics, such as age and sex distributions and stroke distribution by type, were similar across all 3 countries.

There were significant differences in stroke recanalisation treatment across the Baltic states. Lithuania had the highest number of EVTs performed, with 748 cases (8.6% of all strokes), followed by Estonia with 222 cases (7.7%). Latvia had the lowest rate, with only 217 procedures performed, accounting for just 3.7% of stroke cases. In contrast, Estonia had the highest rate of IVT. In Estonia, 839 (29.0%) stroke patients received IVT, compared to 974 (16.8%) in Latvia and 1284 (14.8%) in Lithuania (*P* < .001).

Mortality rates following ischaemic stroke varied significantly among the Baltic States, with Estonia demonstrating better outcomes compared to Lithuania and Latvia. At hospital discharge, ischaemic stroke mortality was lowest in Estonia (9.6%) and highest in Latvia (15%), while intracerebral haemorrhage mortality was lowest in Lithuania at 24.9%, with Estonia at 30.3% and Latvia at 37% (*P* < .001 for both comparisons). Thirty-day mortality rates further highlight these disparities, with persistent significant differences between the 3 countries. Notably, Latvia reported the highest 30-day mortality after intracerebral haemorrhage among European countries submitting National Health Insurance Fund data to SAP-E SST—an alarming 51%.^21^

## Discussion

Our study highlights some significant inter-country differences in stroke care systems across the Baltic states. Firstly, access to stroke treatment varied across countries, with Lithuania having a significantly higher proportion of citizens with access to a stroke-ready hospital within 1 h. Secondly, Estonia had the highest proportion of stroke unit care admissions, nearly twice that of its larger neighbours, as well as the highest overall recanalisation therapy rates. Finally, Estonia consistently demonstrated lower in-hospital and 30-day mortality compared to Lithuania and Latvia for ischaemic stroke patients. These differences suggest potential disparities in access to advanced stroke treatment, which may be influenced by differing national legislation, in-hospital protocols, healthcare infrastructure, emergency response efficiency and general health literacy. Furthermore, we discuss the possible organisational factors contributing to the inter-country differences. For a structured comparison of stroke care systems, see Table 2.

**Table 2:** Structured comparison of stroke system characteristics across the 3 Baltic states in 2022.

Parameter	Lithuania	Latvia	Estonia
**Number of ESO certified stroke centres or stroke units^a^**	0	2	3
**National legislation that all patients with an acute onset of stroke must be transported to the nearest stroke-ready hospital**	Yes	Yes	Yes
**National legislation that stroke can only be diagnosed in stroke-ready hospitals**	Yes	No	No
**National legislation that all patients with an acute onset of stroke must be treated in dedicated stroke units**	No	Yes	Yes
**Universal use of EVT in the extended treatment window**	Yes	Yes	Yes
**Universal use of IVT in the extended treatment window**	No	Partly	Yes
**Use of tenecteplase for IVT^a^**	Yes	Yes	No
**Universal use of CTA in the acute stroke patient population**	No	Partly	Yes
**National stroke care quality monitoring programme**	Yes	No	Yes
**Consistent RES-Q registry participation**	Partly	Partly	Yes
**DOAC reimbursement for primary stroke prevention**	Partly	Partly	Yes
**National telestroke coverage**	No	No	Yes
**Prioritised inpatient poststroke rehabilitation**	Yes	Partly	Yes
**Follow-up at 3–6 months after the incident stroke is provided to at least 90% of patients**	No	No	No

Abbreviations: DOAC, direct oral anticoagulants; IVT, intravenous thrombolysis; RES-Q, Registry of Stroke Care Quality.

^a^Data for December 2025.

The higher hospitalised stroke incidence observed in Latvia and Lithuania, compared with Estonia, may be indicative of differences in primary and secondary stroke prevention (eg, stroke risk factor distribution and medication adherence), rather than due to differences in healthcare infrastructure.^22^ Demographic characteristics, including age and sex distributions and stroke type proportions, were similar across countries, suggesting that population structure alone is unlikely to explain the observed differences in hospitalised stroke incidence.

Although we showed that Lithuania has a significantly higher number of citizens with access to a stroke-ready hospital within 1 h, a previous registry-based study had demonstrated that Estonia had a substantially higher percentage of patients arriving at a stroke-ready hospital within 60 min of symptom onset (23.1%), followed by Lithuania (14.2%) and Latvia (8.7%).^22^ This discrepancy highlights that geographical proximity does not automatically translate into timely hospital arrival.

Public awareness and health-seeking behaviour may be one factor influencing the time from onset to hospital arrival. Population-level education campaigns and cultural differences in health system utilisation might partly explain Estonia’s higher proportion of early presenters.^23^ Additionally, regional disparities in EMS resources may also play a role. This highlights the importance of complementing geographic accessibility analyses with real-world performance indicators when evaluating the effectiveness of stroke networks.

According to national legislation, all patients with an acute onset of stroke in the Baltic states must be transported to the nearest stroke-ready hospital. However, Estonia stands out by having a formal nationwide agreement between the Ministry of Social Affairs, the Health Insurance Fund, the Union of Medical Emergency and all acute care hospitals, stipulating that every stroke patient should be managed in dedicated stroke units or stroke centres, with expanded access to post-stroke rehabilitation.^12^ Although Latvia has similar national legislation, most stroke units are located within local hospitals that are not stroke-ready, and this legislation is poorly enforced. No such legislation exists in Lithuania. This framework likely explains why Estonia has the highest proportion of patients treated in stroke units and illustrates how a supportive legal and policy environment can be instrumental in achieving and sustaining quality-of-care standards.

International guidelines recommend an independent stroke centre certification by an external body.^24^ In addition, SAP-E aims that 90% or more of stroke patients should be treated in a stroke unit as the first level of care.^6^ As of December 2025, there were 3 ESO-certified stroke centres and stroke units in Estonia and 2 in Latvia. In contrast, none of the stroke-ready hospitals in Lithuania have received ESO stroke centre or stroke unit certification to date.^25^

In Lithuania and Latvia, a stroke-ready hospital is required, by law, to operate a dedicated stroke unit and provide 24/7 on-call neurologist coverage. Lithuania’s 2014 ministerial orders include additional accreditation criteria^26^^,^^27^; however, enforcement is weak. To date, none of the stroke-ready hospitals in either country have ever had their stroke centre status revoked for non-compliance. There are no special requirements for stroke units in Estonia; however, the 6 stroke-ready hospitals have accreditation and receive reimbursement for 24/7 acute neurological services, which include acute stroke management.

The use of IVT in the extended treatment window (up to 9 h from symptom onset) was adopted by most CSCs and PSCs in Estonia for the management of wake-up strokes following the publication of the WAKE-UP trial in 2018.^28^ Furthermore, 1 Estonian CSC and 1 PSC have extended this approach beyond 9 h in cases of unknown onset stroke, guided by MRI-based selection criteria. Although thrombolytic treatment of acute ischaemic stroke patients in the extended window is not part of the routine clinical practice in all of the Latvia and Lithuania stroke centres yet, the guidelines are planned to be updated in 2026. The limited use of extended treatment window in Lithuania and Latvia might partly explain their lower IVT rates.

The primary challenges to the routine extension of the thrombolytic window across all countries are the limited availability of CTP in many PSCs, as well as the extended window for recombinant tissue plasminogen activator not having been outlined in the Summary of Product Characteristics by the European Medicines Agency (EMA). Therefore, the National Health Insurance Fund has been reluctant to reimburse it for this indication in Lithuania.^29^ By contrast, in Latvia and Estonia, the therapy is reimbursed whenever it is delivered in a stroke unit, regardless of time from stroke onset.

Another difference between the countries is the use of CTA in the acute stroke patient population. As there are no guidelines for the universal use of CTA in acute stroke patients, there is a sizeable inter-hospital protocol variation in each of the Baltic states: some stroke-ready hospitals perform CTA for all acute ischaemic stroke patients, whereas others only in case of a suspected large vessel occlusion, or lack this diagnostic functionality at all. Therefore, recent registry data have shown that having a CTA for an acute ischaemic stroke in Estonia is 3 times more common than in Latvia.^22^ Together with the differences in the geographical stroke network distribution, this might be one of the factors leading to the significant difference in EVT availability between the Baltic states.

Mortality rates following a stroke reveal stark differences among the Baltic states. Latvia has the highest 30-day ischaemic stroke mortality rate at 25%, followed by Lithuania at 20.8%, while Estonia stands out with a significantly lower rate of 15.0%. Several factors may contribute to this disparity, such as a higher proportion of patients treated in specialised stroke centres and almost twice as many ischaemic stroke patients receiving IVT. Additionally, Estonians may benefit from improved management of risk factors and higher adherence to medication for other comorbidities, including better access to DOACs. Moreover, lower NIHSS scores upon hospital admission in Estonia may reflect a less severe stroke patient population, whereas shorter lengths of in-hospital stay in Estonia could indicate more efficient in-hospital processes.^22^ Finally, Estonia’s markedly higher rate of stroke unit admissions, nearly double that of its Baltic neighbours, likely contributes to its more favourable mortality outcomes. This aligns with the SAP-E, which emphasises that organised stroke unit care significantly reduces stroke-related mortality and long-term disability, and recommends that 90% of stroke patients receive care in dedicated stroke units to reduce mortality and improve functional outcomes.^6^^,^^30^^,^^31^

National stroke care quality monitoring programs have been in place in Lithuania since 2014, and in Estonia since 2015.^32^ In addition, in Lithuania and Latvia, participation in stroke quality registries (such as the Registry of Stroke Care Quality, RES-Q and Safe Implementation of Treatments in Stroke, SITS) is encouraged for at least 1 month during the first quarter of each year.^33–35^ Four stroke-ready hospitals in Estonia provide data to the RES-Q registry continuously throughout the whole year. However, this is difficult to enforce as there is a shortage of personnel who can manually enter data into the registry. Therefore, the Lithuanian government has invested in an interactive national stroke database, where nearly real-time automatic key performance indicator analysis could be performed for data contributed by all Lithuanian PSCs and CSCs^14^^,^^36^—a step towards a learning stroke health system.^37^

The strengths of our study include the use of GIS-based accessibility modelling, which enhances the reliability of our reachability analysis. This approach offers a methodological framework that can be applied to other countries assessing stroke care accessibility.

However, our study has several limitations. First, our data were derived from the National Health Insurance Funds rather than from a prospective registry with uniform data collection methods, and are based on hospitalised stroke cases rather than overall stroke incidence. Nevertheless, these databases represent one of the most widely used sources of nationwide stroke data, as they are used both for reimbursing healthcare institutions and for producing official national health statistics. In addition, because claims are designed for reimbursement, case ascertainment and comparability may be affected (ie, coding variation, reimbursement incentives, limited clinical detail, under-capture of privately financed/non-reimbursed care). Accordingly, these findings may also be influenced by unaccounted differences in patient characteristics, stroke severity or residual selection bias. However, all 3 countries employ standardised data collection protocols, comparable multilevel quality control measures and have similar data capture limitations, such as inclusion of recurrent events and possible omission of some prehospital data. Therefore, while cross-country comparisons should be interpreted with caution, they should remain feasible. Another limitation of this study is that patient data were collected for the full calendar year of 2022, whereas the high-resolution population distribution data were obtained from WorldPop data from 2020. However, this difference is expected to be negligible. Finally, several key SAP-E indicators were not available, including door-to-needle time for IVT and door-to-groin time for EVT, as well as secondary prevention measures such as antiplatelet therapy, anticoagulation, lipid-lowering and antihypertensive therapy, dysphagia screening, rehabilitation and functional outcomes assessed by the mRS at 90 days, as these are not currently captured by the National Health Insurance Funds.

## Conclusions

Despite broadly comparable populations and formal SAP-E alignment, the Baltic states exhibit marked differences in stroke access, treatment and outcomes. Estonia demonstrates lower ischaemic stroke mortality, coinciding with higher stroke unit admission rates and higher recanalisation therapy rates. Our findings emphasise the importance of harmonising stroke care standards and expanding access to high-quality services across the region to meet SAP-E targets by 2030, underscoring the need for system design that extends beyond geographic coverage alone.

## Data Availability

The data that support the findings of this study are available in the Stroke Service Tracker Data repository at https://actionplan.eso-stroke.org/national-stroke-data and are available from the corresponding author upon reasonable request.

## Attributions

Population and map data were provided by WorldPop, d-maps and openrouteservice.
